# Inflammation, Autonomic Control, and Adiposity in Adolescents: Links to Early Cardiovascular Risk

**DOI:** 10.3390/life15091450

**Published:** 2025-09-16

**Authors:** Vladimir Micieta, Ingrid Tonhajzerova, Nikola Ferencova, Zuzana Visnovcova

**Affiliations:** 1 Department of Physiology, Jessenius Faculty of Medicine in Martin, Comenius University in Bratislava, 036 01 Martin, Slovakia; vmicieta@gmail.com (V.M.); ingrid.tonhajzerova@uniba.sk (I.T.); 2 Biomedical Centre Martin, Jessenius Faculty of Medicine in Martin, Comenius University in Bratislava, 036 01 Martin, Slovakia; zuzana.visnovcova@uniba.sk

**Keywords:** inflammatory markers, heart rate variability, anthropometric measures, adolescent cardiovascular risk

## Abstract

Cardiovascular diseases (CVDs) are generally associated with adulthood, although the underlying processes may begin in childhood and adolescence. Thus, detecting increased cardiovascular risk in adolescence is essential for prevention. In this cross-sectional study, we comprehensively evaluated the inter-relationships between inflammatory markers, cardiac autonomic control indexed by heart rate variability (HRV), and adiposity measures in healthy adolescents, with sex-stratified analyses. We examined 90 adolescents (55 females; age 15.8 ± 1.5 years; BMI 21.4 ± 3.3 kg/m^2^). We found mixed (positive and negative) associations between inflammatory markers and HRV indices among the entire adolescent group (absolute r range: 0.266–0.395, *p* < 0.05 for all), while only negative associations in the male group, and only positive associations in the female group (absolute r range: 0.373–0.404, *p* < 0.05 for all). Furthermore, predominantly positive associations between inflammatory markers and adiposity measures were found among the adolescent group (absolute r range: 0.298–0.338, *p* < 0.05 for all) and female group (absolute r range: 0.342–0.485, *p* < 0.05 for all), while contrasting negative associations were found in the male group (absolute r range: 0.421–0.497, *p* < 0.05 for all). These associations, representing early pathways to cardiovascular vulnerability, seem sex-dependent, but longitudinal confirmation is required.

## 1. Introduction

Cardiovascular diseases (CVDs) are usually considered diseases of adulthood; however, the burden of CVDs onset can be traced back to early life, particularly to adolescence [1,2,3]. This critical period, beginning with the onset of puberty and lasting into the mid-20s, is characterized by a profound amount of change in all domains of development—physical, cognitive, emotional, and social [4,5]. The complex interplay of biological factors (e.g., rapid growth and development, body composition changes, such as increase in fat mass and lean mass, and the maturation of the cardiovascular system), lifestyle and behavioral factors (e.g., physical activity level, dietary habits, and sleep patterns), socioeconomic status, and environmental exposures (e.g., air pollution, and noise pollution) influences cardiovascular health in adolescents [6,7,8,9,10,11,12]. Therefore, understanding the factors that influence cardiovascular health during this vulnerable age period can help to develop targeted prevention strategies to promote healthy behaviors and mitigate risk factors, reducing the likelihood of developing CVDs in adulthood.

Mounting evidence suggests that systemic and local inflammation have a central role in the development and progression of CVDs [13]. The major underlying cause of CVDs in adulthood is atherosclerosis, a chronic inflammatory condition characterized by the build-up of plaques inside arteries, primarily composed of lipids that induce an inflammatory response [14,15]. The atherosclerotic process starts to develop already during childhood. The early precursors of atherosclerotic plaques, fatty streaks, appear in the inner layer of the aorta from early childhood, while in the coronary arteries, they begin to form during adolescence [16]. Adolescence represents a critical period for atherosclerosis progression, where early risk factors like obesity, hypertension, and dyslipidemia can accelerate the development of plaques in the arteries, highlighting this important developmental age period as a crucial “window of opportunity” for interventions aimed at preventing atherosclerotic CVDs [17]. Endothelial dysfunction as an early marker for atherosclerosis can be detected before the vessels’ structural changes are apparent on angiographic or ultrasonic examination. Normally, the endothelium has anti-inflammatory and antithrombotic properties, regulating the permeability to circulating molecules and the vascular tone through the endothelium-derived vasodilators (e.g., nitric oxide) and vasoconstrictors (e.g., endothelin) [18]. During the initiation of atherosclerosis, monocytes and T cells are recruited to the vessel wall across an intact endothelium. This process is governed by chemotactic cytokines such as monocyte chemotactic protein-1 (MCP1) produced in the subendothelial layer. When the recruited monocytes enter the subendothelial space, they differentiate into macrophages which can polarize into pro-inflammatory M1 macrophages releasing pro-inflammatory cytokines (e.g., interleukin (IL)-1α, IL-1β, IL-6, IL-8, and the tumor necrosis factor (TNF)-α) contributing to the progression of atherosclerosis or into anti-inflammatory M2 macrophages releasing anti-inflammatory cytokines (e.g., IL-4 and IL-10) contributing to the resolution of inflammation and plaque healing [19]. Since the recent evidence highlights the critical role of pro-inflammatory cytokines in all stages of atherosclerosis, from its initiation to progression [20,21], the evaluation of multiple cytokines’ levels in little-studied adolescents can help identify high-risk individuals for future atherosclerotic CVDs development.

Furthermore, the autonomic nervous system (ANS), consisting of two divisions, the parasympathetic nervous system (PNS) and the sympathetic nervous system (SNS), plays a crucial role in the adaptive regulation of inflammatory processes. The inhibitory function of the vagus is important in regulating the immune response as a rapid reflex anti-inflammatory process. More specifically, following an inflammatory response, afferent signals travel via the vagal nerve to the nucleus of the solitary tract, with a subsequent efferent vagal activity leading to the parasympathetic neurotransmitter—acetylcholine release, interacting with α7 subunits containing acetylcholine receptors expressed on macrophages, with consequent inhibition of the pro-inflammatory cytokine synthesis [22,23,24,25]. This mechanism of neural regulation of inflammatory processes is known as the cholinergic anti-inflammatory pathway. On the other hand, the influence of the SNS activity can contextually both increase and decrease inflammatory responses, however in a slower manner than the PNS pathway [26]. In this context, the dysregulation in the PNS-SNS dynamic balance can lead to pro-inflammatory states potentially contributing to the development and progression of CVDs.

Under physiological conditions, the ANS is strongly involved in different mechanisms of the cardiovascular system activity. The generally accepted and validated marker of this autonomic function is heart rate variability (HRV), i.e., continuous “beat-to-beat” oscillations of heart rate around its mean values, indicating a healthy and adaptive organism [27]. Therefore, HRV analysis represents a valuable non-invasive tool for the detection of discrete abnormalities in cardiac autonomic regulation. The short-term HRV is routinely analyzed by a linear (spectral) method quantifying the high-frequency component that primarily reflects parasympathetic (vagal) activity and the low-frequency components mediated by both parasympathetic and sympathetic inputs [28]. Given the physiological relation between inflammatory processes and predominantly vagal activity via the cholinergic anti-inflammatory pathway, the higher vagally mediated HRV is expected to be associated with decreased levels of inflammation. Although predominant findings support this idea in healthy adults as well as patients with cardiovascular disease, several studies have also reported a positive association between HRV indices and inflammatory markers [26]. Data on the relationships between inflammatory markers and HRV in the adolescent population are almost non-existent. In this context, a recent study found that resting HRV moderated interpersonal stress–inflammation associations only among adolescents with low resting HRV; however, this significant HRV moderation disappeared after multiple-testing correction [29].

One of the traditional CVD risk factors—obesity, characterized by chronic low-grade systemic inflammation—is increasingly observed in children and adolescents worldwide. According to the WHO [30], over 390 million children and adolescents aged 5–19 years are overweight, including 160 million who are obese. Childhood obesity data from the European region are alarming, with one in three children being obese. The most used anthropometric parameter for the diagnosis of overweight/obesity is the body mass index (BMI), although additional anthropometric measures, including waist circumference (WC), waist-to-hip ratio (WHR), overall fat percentage, and visceral fat level, can be helpful. Interestingly, the BMI already within the normal range (i.e., from the 50th to 74th percentiles) during adolescence has been associated with increased cardiovascular morbidity and mortality in adulthood. Moreover, adolescent overweight and obesity have been strongly associated with cardiovascular diseases manifested in adulthood, independent of BMI level [31,32]. To sum up, nowadays, the obesity epidemic is one of the most serious health problems affecting individuals of all ages and contributing to the onset of multiple chronic pathological conditions, including inflammatory and cardiovascular adverse complications. Revealing relationships between adiposity, inflammatory markers, and HRV could contribute to a better understanding of obesity as a risk factor for CVDs. However, only a few studies so far have evaluated these direct associations in adolescents [33,34,35,36,37]. It seems that the main causes of CVDs originate in childhood and adolescence; thus, comprehensive research on risk factors and precise pathophysiological mechanisms contributing to CVDs is crucial. However, the literature leaves unanswered questions about the specific pathophysiological patterns that underpin the onset of CVDs in the vulnerable developmental adolescent period. Therefore, we aimed to evaluate the mutual relationships between inflammatory markers, cardiac autonomic control indexed by HRV, and anthropometric measures in healthy adolescents. We hypothesize sex-dependent differences among “inflammation-cardiovagal control-adiposity” in the adolescent age period.

## 2. Materials and Methods

### 2.1. Participants

The studied group consisted of 90 healthy adolescents (aged: 15.8 ± 1.5 years, BMI: 21.4 ± 3.3 kg/m^2^), which was consequently divided according to sex: females—55 girls (average age: 15.9 ± 1.3 years, BMI: 21.3 ± 3.2 kg/m^2^), and males—35 boys (average age: 15.6 ± 1.7 years, BMI: 21.6 ± 3.6 kg/m^2^) (Table 1). The adolescent participants were recruited from general primary and secondary schools in Martin via informational sessions organized by examiners from the Psychophysiology laboratory at the Department of Physiology and Biomedical Centre Martin, Jessenius Faculty of Medicine in Martin. Firstly, all adolescents who agreed to participate in this study (with the approval of legal guardians when needed) were screened using a health assessment questionnaire. Then, the participants who fulfilled the strict inclusion criteria were examined in the psychophysiological laboratory during October and November within three years (2017–2019) under standard conditions (i.e., a quiet room, minimization of stimuli, in the morning between 8:30 and 11:30 a.m., after a normal breakfast at least 2 h prior to the examination). The inclusion criteria were as follows: adolescence (i.e., age between 10 and 19 years according to WHO), normal blood pressure, at least 8 h of sleep prior to the examination, and no physical activity performed within 24 h before the assessment. Next, the exclusion criteria were as follows: the presence of acute or chronic illness (e.g., diabetes mellitus, infectious diseases, asthma, epilepsy, mental disorders, any type of cancer, etc.), use or abuse of alcohol, drugs, or caffeine, smoking, and any treatment that could influence the autonomic nervous system, specifically HRV (e.g., antidepressants, beta-blockers, calcium-channel blockers, sedatives, analgesics, and anti-seizure medications). This study was approved by the Ethics Committee of Jessenius Faculty of Medicine in Martin, Comenius University in Bratislava (protocol codes EK1970/2017—approval date 25 April 2017, and EK15/2019—approval date 24 April 2019) in accordance with the 1964 Helsinki Declaration and its later amendments. All participants and their parents or legal guardians (for those under 18 years of age) were thoroughly informed about the study procedures and provided written informed consent before the examination.

### 2.2. Protocol

**Figure 1:**
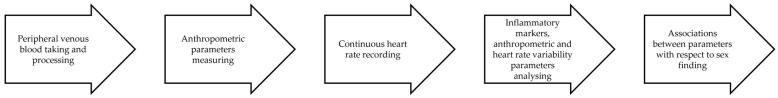
Overall examination protocol.

Firstly, prior to the examination, peripheral venous blood was collected in the morning under fasting conditions and placed into EDTA-containing test tubes. The blood samples were subsequently centrifuged at 2500 rpm for 15 min at 4 °C using a refrigerated centrifuge (Hettich Universal 320R, Tuttlingen, Germany). The resulting plasma was stored at −80 °C until analysis. Afterwards, the examinations were carried out in the psychophysiological laboratory at the Department of Physiology and Biomedical Centre Martin, Jessenius Faculty of Medicine in Martin under standard conditions (i.e., a quiet room, with the minimization of stimuli, in the morning between 8:30 and 11:30 a.m.). The selected anthropometric measures of body composition were measured using InBody 120 (Biospace Co., Ltd., Seoul, Republic of Korea). Subsequently, the participants were seated in an examination armchair, and a sensor was applied for continuous monitoring and recording of heart rate (Polar V800, Polar Electro, Kempele, Finland). Following a 15 min relaxation period to minimize potential stress-related effects, a 6 min resting assessment was recorded (Figure 1).

### 2.3. Data Analysis

#### 2.3.1. Blood Analysis

Five milliliters of fasting peripheral venous blood were collected into EDTA-coated tubes in a sterile manner by an experienced doctor. The whole blood samples were used to assess white blood cell (WBC) counts, including neutrophils (NEU), lymphocytes (LYM), and monocytes (MON) using an automated hematology analyzer (Mindray BC-5500, Guangdong, China). Subsequently, the blood samples were centrifuged at 2500 rpm for 15 min at 4 °C using a refrigerated centrifuge (Hettich Universal 320R, Tuttlingen, Germany). The resulting plasma was stored at −80 °C until further analysis. Selected cytokines—including IL-1α, IL-1β, IL-2, IL-4, IL-6, IL-8, IL-10, TNF-α, IFN-γ, EGF, VEGF, and MCP1—were later quantified using biochip array technology on the Evidence Investigator platform (Randox, Crumlin, County Antrim, Northern Ireland, UK). Each used a high-sensitivity cytokine kit (CTK HS) within the same batch, which comprised test biochip cartridges, calibration standards, assay buffer, conjugate solution, wash solution, and signal reagents (a 1:1 mixture of luminol and peroxide). Quality control samples (CTK HS controls) were utilized to validate each run, and a nine-point calibration curve was conducted for every assay batch in accordance with recommended protocols. Following the addition of 100 μL of plasma samples into the test biochip cartridge, the analytes in the sample interact with the specific immobilized ligands on the biochip surface. The extent of this binding is subsequently assessed through a chemiluminescent signal and quantified using biochip array technology via a Charge-Coupled Device camera and imaging system integrated into the Evidence Investigator platform. All measurements were performed using the same methods, by qualified researchers, and within the same laboratories at the Department of Physiology and Biomedical Centre Martin of the Jessenius Faculty of Medicine in Martin.

#### 2.3.2. Heart Rate Variability Parameters

At the beginning, resting R-R interval time series were recorded for 6 min using the Polar V800 device (Polar Electro, Kempele, Finland) at a sampling frequency of 1000 Hz. Then, all records were carefully checked, and artifacts were removed. The first 30 s of each recording were not used in the analysis. Consequently, artifact-free 5 min segments of the R-R interval data were selected for analysis. In the time-domain analysis, mean heart rate (HR, bpm), standard deviation of successive R-R intervals (SDNN, ms), the root mean square of successive differences (rMSSD, ms), and the percentage of successive R-R intervals differing by more than 50 ms (pNN50, %) from the R-R interval time series were assessed. While parameter SDNN informs about overall variability, both rMSSD and pNN50 parameters reflect cardiac parasympathetic modulation [28,38]. In the spectral domain analysis, R-R interval time series were resampled using cubic spline interpolation at a frequency of 4 Hz. Detrending was subsequently performed using a smoothing parameter (Λ = 500), as described by Tarvainen et al. [39]. Spectral power in the very-low-frequency band (VLF-HRV; 0.0033–0.04 Hz), the low-frequency band (LF-HRV; 0.04–0.15 Hz), and the high-frequency band (HF-HRV; 0.15–0.40 Hz) was estimated using an autoregressive model with a Burg periodogram [40,41]. VLF rhythm reflects, at least in part, an intrinsic cardiac rhythm fundamental to maintaining physiological stability and overall well-being [28,42]. Next, the interpretation of the LF is controversial: while several studies suggest that LF power at rest is predominantly mediated by the PNS [43], other studies pointed to resting LF rhythm as a result of baroreflex activity mediated by both parasympathetic and sympathetic activity [28,44,45]. Finally, the HF rhythms primarily reflect respiratory-linked heart rate fluctuations, indicating cardiac vagal control [46].

#### 2.3.3. Anthropometric Parameters

The selected anthropometric measures of the body composition—BMI, WHR, WC, visceral fat level, and overall fat percentage—were analyzed using InBody 120 (Biospace Co., Ltd., Seoul, Republic of Korea) with reasonable measurement accuracy [47]. The InBody 120 examination uses the Bioelectrical Impedance Analysis, which sends a low-frequency electrical current through the body to measure the impedance (resistance) of different tissues with higher water content (e.g., muscles) that conduct electricity more easily, resulting in lower impedance. Conversely, fat tissue has higher impedance because it is less conductive. The InBody 120 measures this resistance across five body segments (arms, legs, and trunk), providing a detailed body composition analysis. During the examination, the examinee must maintain proper posture to obtain accurate test results—since the examination proceeds only during good electrical contact, the heels need to cover the rear sole electrodes while the foot soles cover the front sole electrodes; and the hand electrodes should be held so that the 4 fingers wrap the surface of the bottom hand electrode, and the thumb is placed on the oval electrode. The arms should be kept straight during examination. The whole examination lasts approximately 15 s. Almost all participants included in the study were normal-weight according to the sex- and age-specific international BMI standards for assessing weight status in children and adolescents, defined by Cole and Lobstein [48].

#### 2.3.4. Statistical Analysis

Data were examined and analyzed using Jamovi version 1.6.9 (Sydney, Australia). The Shapiro–Wilk test was applied to assess the distribution characteristics of the data (parametric vs. non-parametric). The distribution of individual data is described in the Supplementary Materials. Due to substantial inter-individual variability, the spectral power values of HRV parameters (VLF-HRV, LF-HRV, and HF-HRV) were logarithmically transformed. The associations between inflammatory markers (WBC, NEU, LYM, MON, IL-1α, IL-1β, IL-2, IL-4, IL-6, IL-8, IL-10, TNF-α, IFN-γ, EGF, VEGF, and MCP1) and HRV parameters (HR, SDNN, pNN50, rMSSD, lnVLF-HRV, lnLF-HRV, and lnHF-HRV), between inflammatory markers and selected anthropometric measures (BMI, WHR, WC, visceral fat level, and overall fat percentage), and between HRV parameters and anthropometric measures were analyzed using Spearman’s rank-order correlation test. A value of *p* < 0.05 (two-tailed) was considered statistically significant. Given the number of correlations, findings are interpreted as exploratory. The Benjamini–Hochberg (BH) adjustment of *p*-values (pBH; at q = 0.10 within each family/stratum) was applied to account for the false discovery rate and control the family-wise error. Results were deemed statistically significant only when all three of the following criteria were simultaneously satisfied: *p* < 0.05, pBH < 0.05, and *p* < pBH. Additionally, sex differences (i.e., between groups comparison) using Mann–Whitney U test for nonparametric distributed data, and the Welch’s *t*-test for normally distributed data, with effect sizes assessed by Cohen’s d and binomial regression analysis to eliminate confounders, were evaluated and included in the Supplementary Materials.

## 3. Results

### 3.1. Correlations Between Inflammatory Markers and HRV, Inflammatory Markers and Anthropometric Parameters, and Anthropometric Parameters and HRV

#### 3.1.1. Whole Group

Correlation analysis revealed several significant associations between inflammatory markers, cardiac autonomic function, and body composition in healthy adolescents. Higher WBC and NEU counts were positively associated with elevated HR (r = 0.285, *p* = 0.007, pBH = 0.0313; r = 0.322, *p* = 0.002, pBH = 0.0125, respectively). Moreover, increased NEU count correlated with reduced SDNN (r = −0.266, *p* = 0.012, pBH = 0.0500) and lnVLF-HRV (r = −0.395, *p* = 0.009, pBH = 0.0375). On the other hand, the level of IL-8, known as a pro-inflammatory cytokine, was positively associated with lnVLF-HRV (r = 0.353, *p* = 0.005, pBH = 0.0250). With respect to inflammation–body composition interactions, increased NEU count was associated with greater visceral fat level (r = 0.338, *p* = 0.002, pBH = 0.0063) and higher overall fat percentage (r = 0.316, *p* = 0.003, pBH = 0.0188). Higher levels of IL-10, known as an anti-inflammatory cytokine, was associated with lower BMI (r = −0.298, *p* = 0.012, pBH = 0.0438), suggesting its potential protective metabolic role. These findings suggest that subtle inflammatory activity is linked to alterations in cardiac autonomic and adiposity profiles already in a population of healthy adolescents, underscoring the importance of monitoring inflammatory and autonomic markers in youth, as they may provide early signals of cardiovascular risk later in life. No significant correlations were found between the remaining inflammatory markers, HRV indices, or anthropometric measures. A summary of all significant associations is provided in Table 2.

#### 3.1.2. Female Group

In a sample of healthy adolescent females, correlation analysis revealed several significant associations between inflammatory markers, cardiac autonomic function, and body composition. Similarly to the whole adolescent group, higher WBC count was significantly associated with elevated HR (r = 0.373, *p* = 0.005, pBH = 0.0136). Level of TNF-α, known as a pro-inflammatory cytokine, was also positively associated with HR (r = 0.389, *p* = 0.011, pBH = 0.0455). These results suggest sex-specific associations between a low-grade inflammatory activity and cardiac autonomic alterations in healthy adolescent females, potentially indicating sympathetic overactivity and/or reduced vagal activity in response to systemic inflammatory state. On the other hand, a higher level of MCP1 was positively associated with markers of cardiovagal regulation, i.e., with increased rMSSD (r = 0.404, *p* = 0.007, pBH = 0.0182), lnLF-HRV (r = 0.401, *p* = 0.008, pBH = 0.0227), and lnHF-HRV (r = 0.396, *p* = 0.009, pBH = 0.0272). This finding may reflect a regulatory or adaptive role of MCP1 on autonomic function in healthy female adolescent individuals. With respect to inflammation–body composition interactions, higher WBC count was associated with greater overall fat percentage (r = 0.342, *p* = 0.011, pBH = 0.0409), and higher level of IL-6 known predominantly as a pro-inflammatory cytokine was associated with increased WHR (r = 0.485, *p* = 0.001, pBH = 0.0045), WC (r = 0.392, *p* = 0.010, pBH = 0.0318), visceral fat level (r = 0.462, *p* = 0.003, pBH = 0.0091), and overall fat percentage (r = 0.392, *p* = 0.010, pBH = 0.0364) underlying the existence of low-grade inflammation–adiposity relationship already in non-obese, healthy adolescent females. On the other hand, the level of IL-1α was inversely associated with overall fat percentage (r = −0.374, *p* = 0.015, pBH = 0.0500), which may reflect a potential sex-dependent anti-adipogenic role of IL-1 signaling in adolescent females. No other significant correlations were observed between the remaining inflammatory markers, HRV parameters, or anthropometric measures. All significant associations are summarized in Table 3.

#### 3.1.3. Male Group

In healthy adolescent males, correlation analysis revealed sex-specific interactions between immune function, cardiovascular regulation, and adiposity status. Higher WBC count was significantly associated with reduced lnLF-HRV (r = −0.386, *p* = 0.024, pBH = 0.0375). Similarly, elevated NEU count was inversely correlated with reduced SDNN (r = −0.391, *p* = 0.022, pBH = 0.0250). Elevated level of IL-10, an anti-inflammatory cytokine, was associated with lower parasympathetic activity as indicated by reduced pNN50 (r = −0.421, *p* = 0.023, pBH = 0.0333) and lnHF-HRV (r = −0.438, *p* = 0.018, pBH = 0.0208). Furthermore, several inflammatory markers also showed inverse associations with measures of adiposity in adolescent males. IL-1β, IL-2, and IL-10 levels were negatively correlated with BMI (r = −0.464, *p* = 0.011, pBH = 0.0125; r = −0.450, *p* = 0.014, pBH = 0.0167; r = −0.497, *p* = 0.006, pBH = 0.0083, respectively), and higher IL-1β and IL-6 were correlated with lower WHR (r = −0.421, *p* = 0.023, pBH = 0.0292; r = −0.421, *p* = 0.026, pBH = 0.0417, respectively). These inverse findings indicate the complexity of the inflammation-HRV and inflammation–body composition relationship in healthy adolescents with respect to sex. No other significant correlations were found between the remaining inflammatory markers, HRV indices, or anthropometric parameters. All significant associations are summarized in Table 4.

## 4. Discussion

Generally, inflammatory markers, HRV indices, and adiposity measures indicating inter-related biological systems may offer valuable insight into the early pathomechanisms underlying the CVDs development. This is the first study comprehensively revealing these triangular relationships in the rarely studied critical developmental age period–adolescence.

First of all, this study found important interactions between inflammatory markers and cardiac autonomic control indexed by HRV already during adolescence. The whole adolescent group showed positive correlations between mean HR and WBC, NEU, lnVLF-HRV, and IL-8 (i.e., a chemoattractant of neutrophils, [49]), and negative associations between SDNN and NEU, lnVLF-HRV, and NEU. Previous studies evaluating relationships between inflammation and HRV have been primarily focused on the adult population. The majority of these studies generally reported the inverse associations between the vagally mediated HRV indices (e.g., HF-HRV) and pro-inflammatory markers (e.g., C-reactive protein, WBC), indicating an important role of the vagally mediated pathway in the adaptive regulation of inflammatory processes in adults [26,50,51,52]. Recent studies regarding adolescents found a negative correlation between HF-HRV and IL-6, and a positive correlation between LF-HRV and IL-6 [53]; negative associations between rMSSD, HF-HRV, cardiac vagal index, and soluble IL-6 receptor (sIL6R), while positive associations between LF/HF, cardiac sympathetic index, and sIL6R [54]. A previous study reported negative associations between HF-HRV, LF-HRV, and IL-6, CRP [55]. With respect to sex, adolescent females in our study showed positive associations between HRV indices and inflammatory markers, i.e., HR positively correlated with WBC and TNF-α; and rMSSD, lnHF-HRV, and lnLF-HRV positively correlated with MCP1. Adolescent males showed a slightly different pattern: SDNN negatively correlated with NEU count, lnLF-HRV with WBC count, and pNN50, lnHF-HRV with IL-10 (i.e., an anti-inflammatory cytokine modulating inflammatory responses through the regulation of the activity of Th1 cells, monocytes, and macrophages, and through the suppression of the release of the pro-inflammatory cytokines [56,57]). Based on our findings, HRV-inflammation relationships seem to be sex-driven. Since sex-dependent differences in HRV and inflammatory responses can be influenced by neural and hormonal regulation [58,59], further studies are needed to precisely define the subtle alterations in these relationships, especially during adolescence.

Secondly, it is well-known that the body composition can modulate both inflammatory and cardiac autonomic control. Adipose tissue acting as an endocrine organ produces various inflammatory markers; therefore, excess body fat, in particular visceral fat, can significantly contribute to inflammation [60,61]. Positive associations between anthropometric and pro-inflammatory markers were found in adults [62,63]. With respect to the body composition-inflammation relationship during adolescence, the studies so far are focused predominantly on the overweight/obese probands. Specifically, fat mass and visceral adipose tissue were positively correlated with WBC count in overweight/obese university students [35]. Obese adolescents also showed an increase in the systemic immune-inflammation index [36]. Further, body composition indices were positively associated with levels of a high-sensitive C-reactive protein (hsCRP) and TNF-α in prepubertal as well as pubertal children [37]. Interestingly, markers of inflammation, namely IL-6 and hs-CRP, were significantly associated with BMI already in 6-year-old children, with the obese ones showing higher levels of inflammation [64]. Our study revealed positive associations between visceral fat level and NEU, overall fat level and NEU, and negative associations between BMI and IL-10 in the whole adolescent group. These findings indicate that higher levels of visceral and overall fat mass were linked to higher levels of NEU count, and higher BMI with lower levels of anti-inflammatory cytokine, IL-10. This pattern is maintained in adolescent females (i.e., positive associations between adiposity and inflammatory markers, namely positive correlations between WC and IL-6, visceral fat level and IL-6, overall fat level and WBC, IL-6, and between WHR and IL-6, and negative correlation between overall fat level and IL-1α), while adolescent males showed the opposite pattern (i.e., negative associations between adiposity and inflammatory markers, namely negative correlations between BMI and IL-1β, IL-2, IL-10, and between WHR and IL-1β IL-6). These findings pointed to sex-dependent body composition–inflammation relationships. In this context, several studies reported the inverse association between pro-inflammatory markers and testosterone, already in young males (e.g., [65,66,67]). The multidirectional relationship between testosterone and inflammation can be significantly influenced by the adipose tissue, which can represent an eminent source of pro-inflammatory cytokines. Adipose tissue increases the activity of aromatase, i.e., the enzyme converting testosterone to estradiol, and this conversion directly inhibits the hypothalamic–pituitary-gonadal axis, leading to a decrease in the production of testosterone. Further, visceral fat as an active secretory tissue can produce pro-inflammatory cytokines, including IL-1β, IL-6, and TNF-α, adipokines, and other biochemical modulators, which can contribute to systemic and peripheral vascular inflammation. On the other hand, an increased testosterone level and decreased estradiol level (e.g., via aromatase inhibitors) can reduce inflammation, leading to a higher risk of several pathological conditions [68]. None of the anthropometric measures correlated with IL-10 in adolescent females, while BMI negatively correlated with this anti-inflammatory cytokine (i.e., IL-10) in adolescent males. This finding is in accordance with studies revealing a positive correlation between testosterone and IL-10, and negative correlations between body fat and both testosterone and IL-10 [69]. Testosterone levels could play an important role in body composition-inflammation relationships; however, future studies are needed to confirm this assumption.

With respect to adiposity measures and HRV, previous studies reported negative associations between vagally mediated HRV parameters and body fat mass, predominantly in overweight/obese children and adults [70,71,72]. In other words, individuals with higher body fat mass tend to have decreased cardiovagal control, contributing thus to increased cardiovascular risk. The majority of studies have used BMI for evaluating the weight status/body composition-ANS relationships. It is important to note that BMI does not differentiate between fat mass and fat-free mass, or body fat distribution. Therefore, more sophisticated approaches should be used for evaluating the reflection of weight status/body composition on cardiac autonomic regulation. In this context, increased visceral adiposity (not BMI) has been associated with a decrease in vagally mediated HRV indices in children [73] and adults [74], likely due to the pro-inflammatory nature of visceral fat [75,76]. Our study revealed no associations between evaluated anthropometric measures and HRV indices. In the whole adolescent group, neither in adolescent males nor in adolescent females alone. It is important to note that the adiposity indices of almost all probands included in our study were within the age-related normal range. On the other hand, a recent study found negative associations between vagally mediated HRV parameters (rMSSD, pNN50) with several adiposity measures, such as total fat, percent body fat, trunk fat, android fat, gynoid fat, visceral adipose tissue, and subcutaneous abdominal adipose tissue, in adolescents with BMI ranging from normal weight to obesity [33]. Moreover, adolescent probands showed positive associations between visceral adipose tissue and LF-HRV, LF/HF, and negative ones with HF-HRV, indicating a negative influence of increased adiposity on cardiac autonomic functioning [33]. A previous study reported negative associations between increased levels of adiposity and cardiac autonomic functioning indexed by HRV, as well as vascular function in early adolescence [34]. Interestingly, adiposity measures such as BMI and WC were associated with lower vagally mediated HRV already during childhood; children with obesity and low HRV showed altered anthropometric, biochemical, and cardiovascular profiles when compared to children with obesity and normal HRV or controls [77]. The question “normal-weight-overweight-obesity-severe obesity” is still open, requiring further research.

### 4.1. Strengths and Limitations

Strengths of the study include standardized morning assessments; simultaneous evaluation of multiple cytokines (by the same methods, within one batch, within the same laboratory), HRV indices, and anthropometric measures; and sex-stratified analyses.

The study also has several limitations which need to be addressed including the cross-sectional design (precluding causal inference), single-center setting, moderate sample size that limits power for small effects, particularly in sex-stratified analyses, potential recruitment bias, not addressing confounding factors such as socioeconomic status, multiple testing (addressed as exploratory with planned false discovery rate control), and lack of longitudinal data. Longitudinal studies across adolescence with repeated autonomic and inflammatory phenotyping and precise measures of body fat distribution are needed to delineate causal pathways and modifiable targets.

### 4.2. Clinical Implications

This study provides several important insights into clinical implications and future perspectives. Sex-related variations in the associations between inflammatory and HRV indices, as well as between inflammatory and adiposity measures, are already present in healthy adolescents. Thus, early lifestyle interventions enhancing vagal activity associated with a healthy lifestyle, such as a healthy diet, regular physical activity, sleep hygiene, and stress management, leading to reducing visceral adiposity, can promote favorable cytokine profiles.

## 5. Conclusions

Our findings revealed distinct, sex-specific associations between inflammatory markers and HRV indices and between inflammatory markers and body composition measures in adolescents. It could illuminate early pathways leading to higher cardiovascular vulnerability, but longitudinal confirmation is required.

## Figures and Tables

**Table 1:** Basic characteristics of the study cohort.

Parameters	Whole Group	Females	Males
*n*	90	55	35
Age (years)	15.8 ± 1.5	15.9 ± 1.3	15.6 ± 1.7
BMI (kg/m^2^)	21.4 ± 3.3	21.3 ± 3.2	21.6 ± 3.6
HR (bpm)	63.3 ± 11.5	64.6 ± 10.9	62.0 ± 12.4
SBP (mmHg)	112.0 ± 15.8	115.0 ± 16.6	109.0 ± 14.0
DBP (mmHg)	69.2 ± 9.8	70.3 ± 9.8	67.4 ± 9.6
Students of primary school (aged from 10 to 15 years)	35	20	15
Students of secondary school (aged from 16 to 19 years)	55	35	20

BMI—body mass index, HR—heart rate, SBP—systolic blood pressure, DBP—diastolic blood pressure. Data are expressed as count, or mean ± SD.

**Table 2:** Correlation analysis between inflammatory markers, HRV, and anthropometric parameters in the whole group—only significant associations.

Correlations WHOLE GROUP	r-Spearman’s Rank-Order Coefficient	95% Confidence Interval		*p*-Value	pBH-Value
		Lower Limit	Upper Limit		
Inflammatory markers and HRV					
WBC–HR	0.285	0.083	0.46	0.007	0.0313
NEU–HR	0.322	0.12	0.50	0.002	0.0125
NEU–SDNN	−0.266	−0.45	−0.062	0.012	0.0500
NEU–lnVLF-HRV	−0.395	−0.56	−0.20	0.009	0.0375
IL-8–lnVLF-HRV	0.353	0.16	0.52	0.005	0.0250
Inflammatory markers and anthropometric parameters					
NEU—Visceral fat	0.338	0.14	0.51	0.002	0.0063
NEU—Overall fat	0.316	0.12	0.49	0.003	0.0188
IL-10—BMI	−0.298	−0.48	−0.097	0.012	0.0438

WBC—white blood cells, NEU—neutrophils, IL—interleukin, HR—heart rate, SDNN—standard deviation of R-R intervals, lnVLF-HRV—spectral power in the very low-frequency band of the heart rate variability, BMI—body mass index, and BH—Benjamini–Hochberg correction of *p*-value. The results are considered statistically significant correlations if the following conditions are met at the same time: *p* < 0.05, pBH < 0.05, and *p* < pBH.

**Table 3:** Correlation analysis between inflammatory markers, HRV, and anthropometric parameters in the female group—only significant associations.

Correlations FEMALE GROUP	r-Spearman’s Rank-Order Coefficient	95% Confidence Interval		*p*-Value	pBH-Value
		Lower Limit	Upper Limit		
Inflammatory markers and HRV					
WBC–HR	0.373	0.12	0.58	0.005	0.0136
TNF-α–HR	0.389	0.14	0.59	0.011	0.0455
MCP1–rMSSD	0.404	0.16	0.60	0.007	0.0182
MCP1–lnLF-HRV	0.401	0.15	0.60	0.008	0.0227
MCP1–lnHF-HRV	0.396	0.15	0.60	0.009	0.0272
Inflammatory markers and anthropometric parameters					
WBC—Overall fat	0.342	0.084	0.56	0.011	0.0409
IL-6—WHR	0.485	0.25	0.66	0.001	0.0045
IL-6—WC	0.392	0.14	0.60	0.010	0.0318
IL-6—Visceral fat	0.462	0.22	0.65	0.003	0.0091
IL-6—Overall fat	0.392	0.14	0.60	0.010	0.0364
IL-1^α^—Overall fat	−0.374	−0.58	−0.12	0.015	0.0500

WBC—white blood cells, IL—interleukin, MCP1—monocyte chemoattractant protein-1, TNF-α—tumor necrosis factor-alpha, WC—waist circumference, WHR—waist to hip ratio, HR—heart rate, rMSSD—root mean square of the successive differences in the R-R intervals duration, lnLF-HRV—spectral power in the low-frequency band of the heart rate variability, lnHF-HRV—spectral power in the high-frequency band of the heart rate variability, and BH—Benjamini–Hochberg correction of *p*-value. The results are considered statistically significant correlations if the following conditions are met at the same time: *p* < 0.05, pBH < 0.05, and *p* < pBH.

**Table 4:** Correlation analysis between anthropometric parameters, heart rate variability indices, and inflammatory markers in the male group—only significant associations.

Correlations MALE GROUP	r-Spearman’s Rank-Order Coefficient	95% Confidence Interval		*p*-Value	pBH-Value
		Lower Limit	Upper Limit		
Inflammatory markers and HRV					
WBC–lnLF-HRV	−0.386	−0.64	−0.061	0.024	0.0375
NEU–SDNN	−0.391	−0.64	−0.066	0.022	0.0250
IL-10–pNN50	−0.421	−0.66	−0.10	0.023	0.0333
IL-10–lnHF-HRV	−0.438	−0.67	−0.12	0.018	0.0208
Inflammatory markers and anthropometric parameters					
IL-1β—BMI	−0.464	−0.69	−0.15	0.011	0.0125
IL-1β—WHR	−0.421	−0.66	−0.10	0.023	0.0292
IL-2—BMI	−0.450	−0.68	−0.14	0.014	0.0167
IL-6—WHR	−0.421	−0.66	−0.10	0.026	0.0417
IL-10—BMI	−0.497	−0.71	−0.20	0.006	0.0083

WBC—white blood cells, NEU—neutrophils, IL—interleukin, BMI—body mass index, WHR—waist to hip ratio, SDNN—standard deviation of R-R intervals, pNN50—the proportion of R-R50 divided by the total number of R-R, lnLF-HRV—spectral power in the low-frequency band of the heart rate variability, lnHF-HRV—spectral power in the high-frequency band of the heart rate variability, and BH—Benjamini–Hochberg correction of *p*-value. The results are considered statistically significant correlations if the following conditions are met at the same time: *p* < 0.05, pBH < 0.05, and *p* < pBH.

## Data Availability

Data are available upon reasonable request from the corresponding author.

## Supplementary Materials

The additional information regarding sample size calculation, between-sex comparison, and effect of sex on inflammatory, HRV, and anthropometric parameters can be downloaded at: https://www.mdpi.com/article/10.3390/life15091450/s1, Table S1: Pilot cohort to evaluate sample size, coefficient α = 0.05, power (1-β) = 0.90, N = 16, N1 (adolescent females) = 10, N2 (adolescent males) = 6, N2/N1 = 0.60; Table S2: Inflammatory markers; Table S3: HRV parameters; Table S4: Anthropometric parameters; Table S5: Estimated relationships between sex and inflammatory markers in healthy adolescents; Table S6: Estimated relationships between sex and HRV parameters in healthy adolescents; Table S7: Estimated relationships between sex and anthropometric parameters in healthy adolescents.

## Abbreviations

The following abbreviations are used in this manuscript:

ANS	Autonomic nervous system
BMI	Body mass index
CVDs	Cardiovascular diseases
EGF	Epidermal growth factor
HF-HRV	High frequency band of heart rate variability
HR	Heart rate
HRV	Heart rate variability
IFN	Interferon
IL	Interleukin
LF-HRV	Low frequency band of heart rate variability
LPS	Lipopolysaccharide
LYM	Lymphocytes
MCP1	Monocyte chemotactic protein-1
MON	Monocytes
NEU	Neutrophils
pNN50	Proportion of R-R50 divided by the total number of R-R
PNS	Parasympathetic nervous system
rMSSD	Root mean square of the successive differences in the R-R intervals duration
SDNN	Standard deviation of R-R intervals
SNS	Sympathetic nervous system
TNF	Tumor necrosis factor
VEGF	Vascular endothelial growth factor
VLF-HRV	Very low frequency band of heart rate variability
WBC	White blood cells
WC	Waist circumference
WHO	World Health Organization
WHR	Waist-to-hip ratio
