# Supplementary material for: Inflammation, Autonomic Control, and Adiposity in Adolescents: Links to Early Cardiovascular Risk
Source: Life (Basel). 2025 Sep 16;15(9):1450. doi: 10.3390/life15091450 (PMC12471339; doi:10.3390/life15091450)
Supplement: Supplementary file 1 [file life-15-01450-s001.zip › life-3824061-supplementary.pdf]

Supplementary material

Additional statistical analysis

Data were examined and analyzed using Jamovi version 1.6.9 (Sydney, Australia). Firstly, the sample size was generated from a pilot cohort consisted of 16 probands (10 girls, 6 boys). Consequently, the required sample size of 90 probands (55 girls, 35 boys) was calculated by GPower v. 3.1. A basic table of mean ± SD, effect size d, recommended N for the whole group and individually for girls and boys, as well as the actual power is presented in Table S1.

Table. S1 Pilot cohort to evaluate sample size, coefficient α = 0.05, power (1-β) = 0.90, N = 16, N1 (adolescent females) = 10, N2 (adolescent males) = 6, N2/N1 = 0.60

parameter	Mean ± SD – adolescent females	Mean ± SD – adolescent males	Effect size d	N1 - adolescent females	N2 - adolescent males	N (N1+N2)	Actual Power
			Inflammatory markers				
WBC (10^9/L)	7.03±1.86	5.92±1.07	0.732	44	26	70	0.900
NEU (10^9/L)	3.79±1.21	3.08±0.86	0.676	51	31	82	0.903
LYM (10^9/L)	2.42±0.667	2.89±0.506	0.794	38	22	60	0.900
MON (10^9/L)	0.537±0.079	0.600±0.067	0.859	33	19	52	0.903
IL-1α (pg/mL)	0.403±0.298	0.217±0.104	0.833	35	21	56	0.909
IL-1β (pg/mL)	2.28±2.19	1.01±0.617	0.789	39	23	62	0.907
IL-2 (pg/mL)	5.09±3.52	2.66±3.09	0.734	44	26	70	0.902
IL-4 (pg/mL)	3.34±2.19	1.84±0.626	0.931	28	16	44	0.900
IL-6 (pg/mL)	1.31±1.03	1.99±1.05	0.654	55	33	88	0.903
IL-8 (pg/mL)	4.28±1.6	5.66±1.1	1.005	24	14	38	0.901
IL-10 (pg/mL)	0.794±0.109	0.663±0.194	0.833	35	21	56	0.909
TNF-α (pg/mL)	2.54±0.803	2.05±0.714	0.645	56	34	90	0.903
IFN-γ (pg/mL)	0.876±0.237	0.36±0.219	2.261	6	4	10	0.939
MCP1 (pg/mL)	71.6±29.9	94.3±37.5	0.669	53	31	84	0.902
EGF (pg/mL)	12.2±5.34	8.3±6.26	0.670	53	31	84	0.902
VEGF (pg/mL)	13.8±3.54	16.6±4.26	0.715	46	28	74	0.905
			Heart rate variability parameters				
HR (bpm)	64.5±12.1	56.8±10.8	0.671	53	31	84	0.903
SDNN (ms)	46.2±17.9	82.3±53.4	0.906	29	17	46	0.900
pNN50 (%)	13.4±10.8	22.1±15.0	0.666	53	31	84	0.900
rMSSD (ms)	32.9±15.7	47.1±21.1	0.764	41	25	66	0.909
lnVLF-HRV (ms^2)	-1.12±0.456	-0.713±0.511	0.840	34	20	54	0.903
lnLF-HRV (ms^2)	-0.195±0.509	0.277±0.625	0.828	35	21	56	0.906
lnHF-HRV (ms^2)	1.71±0.278	1.94±0.391	0.678	51	31	82	0.904
			Anthropometric parameters				
BMI (kg/m^2)	20.5±2.12	22.3±3.32	0.660	54	32	86	0.902
WHR	0.833±0.033	0.867±0.062	0.685	50	30	80	0.903

WC (cm)	75.7±6.53	79.3±4.12	0.659	54	32	86	0.901
Visceral fat (%)	5.3±2.06	4.07±1.38	0.702	48	28	76	0.900
Overall fat (%)	23.0±5.89	14.0±11.0	1.020	24	14	38	0.908

WBC – white blood cells, NEU – neutrophils, LYM – lymphocytes, MON – monocytes, IL – inter-leukin, TNF-α – tumor necrosis factor-alpha, IFN-γ – interferon-gamma, MCP1 – monocyte chemoattractant protein-1, EGF – epidermal growth factor, VEGF – vascular endothelial growth factor, HR – heart rate, SDNN – standard deviation of R-R intervals, pNN50 – the proportion of R-R50 divided by the total number of R-R, rMSSD – root mean square of the successive differences of the R-R intervals duration, lnVLF-HRV – spectral power in the very low-frequency band of the heart rate variability, lnLF-HRV – spectral power in the low-frequency band of the heart rate variability, lnHF-HRV – spectral power in the high-frequency band of the heart rate variability, BMI – body mass index, WHR – waist to hip ratio, and WC – waist circumference.

Next, the Shapiro–Wilk test was applied to assess the distribution characteristics of the data (parametric vs. non-parametric) (*Whole group:* normally distributed data: overall fat – p = 0.237, lnVLF-HRV – p = 0.057, lnHF-HRV – p = 0.494, MON – p = 0.819, and MCP1 – p = 0.098; not normally distributed data: BMI – p < 0.001, WHR – p < 0.001, WC – p < 0.001, visceral fat – p < 0.001, HR – p = 0.002, SDNN – p < 0.001, pNN50 – p < 0.001, RMSSD – p < 0.001, lnLF-HRV – p = 0.033, WBC – p < 0.001, NEU – p < 0.001, LYM – p = 0.045, IL-1α – p < 0.001, IL-1β – p < 0.001, IL-2 – p < 0.001, IL-4 – p < 0.001, IL-6 – p < 0.001, IL-8 – p < 0.001, IL-10 – p < 0.001, TNFα – p < 0.001, INFγ – p < 0.001, EGF – p < 0.001, and VEGF – p < 0.001; *Adolescent females:* normally distributed data: HR – p = 0.127, SDNN – p = 0.065, lnVLF-HRV – p = 0.225, lnLF-HRV – p = 0.172, lnHF-HRV – p = 0.252, MON – p = 0.789, and MCP1 – p = 0.052; not normally distributed data: BMI – p < 0.001, WHR – p = 0.019, WC – p < 0.001, visceral fat – p < 0.001, overall fat – p = 0.004, pNN50 – p < 0.001, RMSSD – p = 0.007, WBC – p < 0.001, NEU – p < 0.001, LYM – p = 0.009, IL-1α – p < 0.001, IL-1β – p < 0.001, IL-2 – p < 0.001, IL-4 – p < 0.001, IL-6 – p < 0.001, IL-8 – p < 0.001, IL-10 – p < 0.001, TNFα – p < 0.001, INFγ – p < 0.001, EGF – p < 0.001, and VEGF – p < 0.001; *Adolescent males:* normally distributed data: lnVLF-HRV – p = 0.300, lnLF-HRV – p = 0.407, lnHF-HRV – p = 0.984, WBC – p = 0.190, LYM – p = 0.988, MON – p = 0.906, and MCP1 – p = 0.276; not normally distributed data: BMI – p < 0.001, WHR – p = 0.005, WC – p < 0.001, visceral fat – p < 0.001, overall fat – p = 0.002, HR – p = 0.002, SDNN – p = 0.005, pNN50 – p < 0.001, RMSSD – p = 0.002, NEU – p = 0.015, IL-1α – p < 0.001, IL-1β – p = 0.002, IL-2 – p < 0.001, IL-4 – p < 0.001, IL-6 – p < 0.001, IL-8 – p < 0.001, IL-10 – p = 0.011, TNFα – p = 0.027, INFγ – p < 0.001, EGF – p < 0.001, and VEGF – p < 0.001).

Between sex comparisons (males vs. females) were first evaluated using the Mann–Whitney U test for non-normally distributed variables and Welch's t-test for normally distributed data. Subsequently, to account for the false discovery rate and control the family-wise error, the Benjamini–Hochberg (BH) adjustment of p-values (pBH) was applied, which is a widely accepted approach for multiple comparisons. Results were deemed statistically significant only when all three of the following criteria were simultaneously satisfied: p < 0.05, pBH < 0.05, and p < pBH.

To further examine predictors of sex, binomial logistic regression analyses were performed. Sex (male and female) was specified as the dependent variable, and relevant anthropometric, heart rate variability, and inflammatory parameters were entered as independent variables. Model fit was evaluated using likelihood-ratio χ^2 tests, Akaike Information Criterion (AIC). Regression coefficients (Estimate),

standard errors (SE), Wald statistics (Z), and odds ratios (OR) with 95% confidence intervals (CI) were reported. Statistical significance of regression analysis was set at $p < 0.05$ (two-tailed).

Results of the between-sex comparisons

Inflammatory Markers

Statistical analysis revealed significantly lower plasma levels of MCP1 in a female group compared to the male group (68.0 pg/mL vs 83.7 pg/mL, p = 0.001, pBH = 0.0063). No significant changes were found in the remaining cytokines between females and males. All results are summarized in Table S2.

Table S2. Inflammatory markers.

Parameters	Whole group	Females	Males	Cohen´s d	Mean difference	SE difference	95% Confidence interval		p - value	pBH-value
							Lower limit	Upper limit		
WBC (109/L)	6.9 (6.4, 8.0)	7.5 (6.1, 8.5)	6.7 (5.9, 7.3)	0.385	0.440	0.386	-0.190	1.120	0.148	0.050
NEU (109/L)	3.3 (2.8, 4.4)	3.6 (2.9, 4.7)	3.6 (2.7, 3.9)	0.419	0.370	0.337	-0.060	0.870	0.094	0.025
LYM (109/L)	2.5 (2.1, 3.1)	2.5 (2.1, 3.0)	2.6 (2.1, 3.0)	0.021	-0.010	0.133	-0.280	0.260	0.973	0.100
MON (109/L)	0.51 (0.45, 0.63)	0.52 (0.47, 0.64)	0.50 (0.44, 0.62)	0.222	0.030	-0.029	0.088		0.303	0.0625
IL-1α (pg/mL)	0.24 (0.18, 0.35)	0.24 (0.18, 0.41)	0.22 (0.18, 0.32)	0.363	0.060	0.130	-6.60e-5	0.130	0.383	0.075
IL-1β (pg/mL)	1.4 (0.9, 2.2)	1.5 (1.0, 2.7)	1.3 (0.8, 1.9)	0.511	0.520	0.915	0.070	1.170	0.029	0.0125
IL-2 (pg/mL)	2.3 (1.4, 5.0)	2.7 (1.5, 5.3)	2.0 (1.4, 3.8)	0.409	0.370	1.285	-0.330	1.940	0.087	0.0188
IL-4 (pg/mL)	2.2 (1.8, 2.7)	2.3 (1.8, 2.8)	2.0 (1.8, 2.6)	0.331	0.350	0.710	-5.05e-5	0.690	0.124	0.0375
IL-6 (pg/mL)	0.90 (0.65, 1.22)	0.97 (0.69, 1.30)	0.81 (0.61, 1.14)	-0.256	0.070	0.921	-0.160	0.300	0.138	0.0438
IL-8 (pg/mL)	3.7 (2.7, 6.1)	3.8 (2.6, 6.1)	3.7 (2.9, 5.9)	-0.081	-0.070	1.662	-0.960	1.030	0.829	0.0875
IL-10 (pg/mL)	0.87 (0.63, 1.24)	0.84 (0.63, 1.37)	0.91 (0.64, 1.19)	0.247	0.040	0.452	-0.140	0.210	0.841	0.0938
TNF-α (pg/mL)	2.8 (2.2, 3.4)	2.8 (2.0, 3.4)	2.8 (2.3, 3.5)	0.181	-0.070	0.682	-0.520	0.480	0.248	0.0563
IFN-γ (pg/mL)	0.45 (0.28, 0.75)	0.48 (0.29, 0.83)	0.39 (0.28, 0.67)	0.280	0.140	1.974	-0.010	0.280	0.097	0.0313
MCP1 (pg/mL)	74.9 (55.9, 95.4)	68.0 (51.3, 85.6)	83.7 (69.0, 102.0)	-0.569	21.640	8.452	37.640	-6.560	**0.001**	**0.0063**

EGF (pg/mL)	13.9 (7.5, 22.5)	14.5 (7.9, 23.0)	13.0 (6.3, 22.3)	0.138	4.030	4.051	1.000	7.760	0.360	0.0688
VEGF (pg/mL)	12.7 (8.2, 19.8)	13.6 (7.5, 20.5)	11.4 (8.6, 19.5)	0.168	2.150	3.587	-1.860	5.890	0.690	0.0813

WBC – white blood cells, NEU – neutrophils, LYM – lymphocytes, MON – monocytes, IL – interleukin, TNF-α – tumor necrosis factor-alpha, IFN-γ – interferon-gamma, MCP1 – monocyte chemoattractant protein-1, EGF – epidermal growth factor, VEGF – vascular endothelial growth factor, and BH– Benjamini Hochberg correction of p value. Data are expressed as median (IQR). The p-value expresses the comparison between groups. The results are considered statistically significant sex differences if the following conditions are met at the same time: $p < 0.05$, $pBH < 0.05$, and $p < pBH$ (in bold).

Our study revealed sex-related differences in MCP1 levels, an important chemokine that attracts predominantly monocytes to inflammatory sites and is known to be associated with atherosclerotic changes; increased levels of MCP1 were associated with peripheral vascular disease and was considered an independent risk factor in coronary artery disease [1]. In our study, the MCP1 levels were significantly higher in adolescent males compared to adolescent females. The pattern of sexual dimorphism observed in our study regarding MCP1 can be partially explained by sex hormones. Experimental models have proposed the inhibitory effect of estrogen on the MCP1 expression [2], while testosterone enhanced LPS-induced MCP1 expression [3]. However, studies regarding sex differences in MCP1 levels revealed no difference between healthy adult males and females [4] or higher MCP1 levels in males compared to premenopausal females [5]. Further studies are thus needed for a precise understanding of the underlying mechanisms involved in the sex-related differences in MCP1 levels, especially in this critical developmental age period during which the hormonal balance sharply shifts towards an adult state.

HRV Parameters

Statistical analysis revealed no significant changes between females and males in selected HRV parameters. All results are summarized in Table S3.

Table S3. HRV parameters.

**Selected HRV parameters**	**Whole group**	**Females**	**Males**	**Cohen´s d**	**Mean difference**	**SE difference**	**95% Confidence interval**		**p - value**	**pBH-value**
							**Lower limit**	**Upper limit**		
HR (bpm)	63.6 ± 11.5	64.6 ± 10.9	62.0 ± 12.4	0.232	2.667	2.527	-2.357	7.690	0.294	0.0256
SDNN (ms)	62.9 ± 28.3	59.2 ± 23.3	68.8 ± 34.5	-0.341	-9.574	6.131	21.760	2.612	0.122	0.0143
pNN50 (%)	14.9 (4.6, 36.5)	15.6 (4.4, 36.4)	12.3 (5.2, 35.7)	-0.069	-1.440	4.563	10.510	7.630	0.753	0.1000
rMSSD (ms)	37.0 (27.1, 64.4)	38.7 (27.4, 59.1)	35.1 (26.7, 68.6)	-0.136	-3.422	5.485	4.324	7.481	0.534	0.0571

lnVLF-HRV (ms²)	-1.32 ± 0.69	-1.38 ± 0.63	-1.22 ± 0.77	-0.124	-0.053	0.093	-0.238	0.132	0.312	0.0429
lnLF-HRV (ms²)	0.86 ± 0.52	0.84 ± 0.51	0.91 ± 0.54	-0.141	-0.069	0.107	-0.280	0.143	0.548	0.0714
lnHF-HRV (ms²)	2.43 ± 0.49	2.44 ± 0.47	2.40 ± 0.52	0.065	0.033	0.109	-0.184	0.249	0.714	0.0857

HR – heart rate, SDNN – standard deviation of R-R intervals, pNN50 – the proportion of R-R50 divided by the total number of R-R, rMSSD – root mean square of the successive differences of the R-R intervals duration, lnVLF-HRV – spectral power in the very low-frequency band of the heart rate variability, lnLF-HRV – spectral power in the low-frequency band of the heart rate variability, lnHF-HRV – spectral power in the high-frequency band of the heart rate variability, and BH–Benjamini Hochberg correction of p value.. Gaussian distributed data are expressed as mean ± SD; non-parametric data as median (IQR). The p-value expresses the comparison between groups. The results are considered statistically significant sex differences if the following conditions are met at the same time: $p < 0.05$, $pBH < 0.05$, and $p < pBH$ (in bold).

There is increasing evidence of higher vagal control in females compared to males despite greater HR. This sex-related difference has been more pronounced in older participants when compared to the younger ones [6]. This relative vagal dominance in adult females is considered a cardioprotective factor associated with overall greater health [7,8], but the developmental period of the sex difference onset is still discussed. Several studies revealed no sex difference in HR and HRV [9], higher HR and lower HRV in girls compared to boys [10,11], or age-dependent sex differences in HR and HRV [12,13]. However, the most recent meta-analysis concluded higher HR associated with lower vagally-mediated HRV in girls compared to boys [14]. In our study, HR and vagally-mediated HRV indices were slightly higher in adolescent females compared to males (but not significantly) supporting the findings in young adults. From this point of view, a shift from pre-pubertal lower vagal activity to post-pubertal greater vagal activity in girls compared to boys may be related to hormonal changes during this sensitive developmental period. Specifically, changes in female sex hormones estrogen and progesterone associated with pubertal development have an enhancing effect on cardiac vagal function, potentially explaining the change from lower vagal activity in girls to relatively higher vagal activity in women. In boys, the pubertal development associated with rising testosterone levels may shift the relative dominance of vagal activity to decreased vagal activity compared to females in adulthood [14].

Anthropometric Parameters

Between-sex comparison revealed significantly higher percentage of overall fat and visceral fat level in females compared to males (24.4% vs 15.4%, $p < 0.001$, $pBH = 0.020$; 5.9 vs 3.9, $p = 0.006$, $pBH = 0.040$; respectively). No significant changes were found in the remaining parameters. Anthropometric parameters are summarized in Table S4.

Table S4. Anthropometric parameters.

**Parameters**	**Whole group**	**Females**	**Males**	**Cohen´s d**	**Mean difference**	**SE difference**	**95% Confidence interval**		**p - value**	**pBH- value**
							**Lower limit**	**Upper limit**		

BMI (kg/m2)	21.4 ± 3.3	21.3 ± 3.2	21.6 ± 3.6	- 0.100	-0.336	0.733	-1.792	1.120	0.648	0.060
WHR	0.84 ± 0.05	0.84 ± 0.05	0.84 ± 0.05	0.032	0.002	0.011	-0.019	0.023	0.882	0.080
WC (cm)	77.4 ± 9.2	77.5 ± 9.0	77.3 ± 9.5	0.024	0.225	2.009	-3.769	4.219	0.911	0.100
Visceral fat	5.1 ± 3.2	5.9 ± 2.9	3.9 ± 3.3	0.640	1.959	0.688	0.591	3.328	**0.006**	**0.040**
Overall fat (%)	21.0 ± 8.4	24.4 ± 6.7	15.4 ± 8.0	1.140	9.070	1.569	5.952	12.187	**< 0.001**	**0.020**

BMI – body mass index, WHR – waist to hip ratio, WC – waist circumference, and BH–Benjamini Hochberg correction of p value. Data are expressed as mean ± SD; The results are considered statistically significant sex differences if the following conditions are met at the same time: $p < 0.05$, $pBH < 0.05$, and $p < pBH$ (in bold).

In this study, adolescent females showed higher level of visceral and overall fat percentage compared to adolescent males. Adolescent females tend to accumulate more body fat, while adolescent males tend to gain larger amounts of lean mass. The sex-driven differences can also be observed in body fat distribution. Specifically, females tend to accumulate fat peripherally and in hips and males in the waist region [15,16].

The results of the regression analysis

Impact of sex on inflammatory markers in healthy adolescents

A binomial logistic regression was conducted to assess impact of sex on inflammatory markers (WBC, NEU, LYM, MON, IL-1α, IL-1β, IL-2, IL-4, IL-6, IL-8, IL-10, INF-γ, TNF-α, MCP1, EGF, and VEGF) in healthy adolescents. The overall model was statistically significant, $\chi^2(16) = 35.4$, $p = 0.003$, indicating that the predictor set distinguished between sexes better than chance. However, model fit indices suggested modest explanatory power (Deviance = 61.7; AIC = 95.7). Among the predictors, IL-6 (β = 1.18, SE = 0.70, p = 0.089, OR = 3.27, 95% CI [0.84, 12.78]) and VEGF (β = –0.10, SE = 0.06, p = 0.087, OR = 0.91, 95% CI [0.81, 1.01]) showed trends to statistical significance, suggesting possible—but inconclusive sex-related differences. All other markers, including WBC, NEU, LYM, MON, IL-1α, IL-1β, IL-2, IL-4, IL-8, IL-10, IFN-γ, TNF-α, MCP1, EGF, and VEGF, were not significant predictors (Table S5).

Table S5. Estimated relationships between sex and inflammatory markers in healthy adolescents.

Predictor	β Estimate	SE	95% Confidence Interval		Odds ratio	Z	p
			Lower	Upper			
Intercept	3.00883	2.4966	-1.88442	7.9021	20.2638	1.2052	0.228
WBC	2.92880	2.4331	-1.83999	7.6976	18.7052	1.2037	0.229
NEU	-3.59899	2.5207	-8.53947	1.3415	0.0274	-1.4278	0.153
LYM	-3.50142	2.8128	-9.01446	2.0116	0.0302	-1.2448	0.213
MON	-3.14908	3.9138	-10.82002	4.5219	0.0429	-0.8046	0.421
IL-1α	-0.71819	2.8932	-6.38872	4.9523	0.4876	-0.2482	0.804
IL-1β	-0.98376	0.6066	-2.17262	0.2051	0.3739	-1.6218	0.105

IL-2	0.27334	0.2748	-0.26536	0.8120	1.3143	0.9945	0.320
IL-4	-0.86484	0.7638	-2.36179	0.6321	0.4211	-1.1323	0.257
IL-6	1.18427	0.6957	-0.17928	2.5478	3.2683	1.7023	0.089
IL-8	0.12068	0.1588	-0.19052	0.4319	1.1283	0.7601	0.447
IL-10	0.79399	1.0679	-1.29903	2.8870	2.2122	0.7435	0.457
INF-γ	-1.58444	1.3149	-4.16165	0.9928	0.2051	-1.2050	0.228
TNF-α	0.18845	0.5034	-0.79811	1.1750	1.2074	0.3744	0.708
MCP1	0.01986	0.0123	-0.00415	0.0439	1.0201	1.6209	0.105
EGF	0.00115	0.0194	-0.03693	0.0392	1.0012	0.0594	0.953
VEGF	-0.09600	0.0561	-0.20599	0.0140	0.9085	-1.7107	0.087

WBC – white blood cells, NEU – neutrophils, LYM – lymphocytes, MON – monocytes, IL – interleukin, TNF-α – tumor necrosis factor-alpha, INF-γ – interferon-gamma, MCP1 – monocyte chemoattractant protein-1, EGF – epidermal growth factor, and VEGF – vascular endothelial growth factor. A value of p < 0.05 (in bold) was considered statistically significant.

Although the overall model was statistically significant, individual predictors largely did not reach conventional significance levels. Among the most informative predictors, IL-6 showed a positive association with male sex, which aligns with previous studies reporting elevated IL-6 levels in males [17], potentially due to sex hormone modulation of immune responses [18,19]. VEGF was inversely associated with male sex (OR = 0.91, p = 0.087), suggesting a potential sex difference in angiogenic signaling, which is consistent with findings that estrogen promotes VEGF expression in females [20]. The negative but non-significant associations for IL-1β, IL-10, and IFN-γ with male sex also reflect broader sex-based immune differences, where females tend to mount stronger inflammatory and adaptive immune responses [18,21].

Impact of sex on heart rate variability parameters in healthy adolescents

A binomial logistic regression was conducted to determine the impact of sex on HRV parameters (HR, SDNN, pNN50, rMSSD, lnVLF_HRV, lnLF_HRV, and lnHF_HRV) in healthy adolescents. The overall model was not statistically significant, $\chi^2(7) = 8.17$, $p = 0.318$, suggesting that the set of predictors did not reliably distinguish between sexes. Thus, regression analysis revealed, that none of the HRV parameters were significant predictors of sex (Table S6).

Table S6. Estimated relationships between sex and HRV parameters in healthy adolescents.

Predictor	β Estimate	SE	95% Confidence Interval		Odds ratio	Z	p
			Lower	Upper			
Intercept	2.91172	5.3069	5.59e-4	605097.46	18.388	0.5487	0.583
HR	-0.02004	0.0222	0.9384	1.02	0.980	-0.9019	0.367
SDNN	0.03025	0.0195	0.9920	1.07	1.031	1.5475	0.122
pNN50	0.00790	0.0305	0.9495	1.07	1.008	0.2594	0.795
rMSSD	0.00186	0.0298	0.9450	1.06	1.002	0.0625	0.950
lnVLF_HRV	0.77106	3.9420	9.54e-4	4900.90	2.162	0.1956	0.845
lnLF_HRV	-0.94658	3.2835	6.22e-4	242.01	0.388	-0.2883	0.773
lnHF_HRV	-1.78405	1.2720	0.0139	2.03	0.168	-1.4025	0.161

HRV – heart rate variability, HR – heart rate, SDNN – standard deviation of R-R intervals, pNN50 – the proportion of R-R50 divided by the total number of R-R, rMSSD – root mean square of the successive differences of the R-R intervals duration, lnVLF-HRV – spectral power in the very low-frequency band of the heart rate variability, lnLF-HRV – spectral power in the low-frequency band of the heart rate variability, and lnHF-HRV – spectral power in the high-frequency band of the heart rate variability. A value of $p < 0.05$ (in bold) was considered statistically significant.

This study found no significant differences in HRV between males and females, despite the known influence of sex hormones and autonomic nervous system regulation. Previous research has reported mixed findings: some studies observed higher SDNN in males [22] and higher rMSSD or HF-HRV in females [6,23], while others found no consistent sex-related patterns [24]. Although HRV reflects physiological processes that vary by sex, future research should account for additional variables and explore nonlinear or interaction effects to better evaluate HRV as a potential sex-specific biomarker.

Impact of sex on anthropometric parameters in healthy adolescents

A binomial logistic regression analysis was performed to examine the impact of sex to anthropometric parameters (BMI, WHR, WC, visceral fat, and overall fat) in healthy adolescents. The overall model was statistically significant, $\chi^2(5) = 59.50$, $p < 0.001$, indicating that the predictors as a set reliably distinguished between males and females. Model fit indices suggested adequate fit (Deviance = 52.2; AIC = 64.2). Regression analysis revealed that BMI and overall fat emerged as significant predictors of sex. Specifically, higher BMI was associated with greater odds of being male ($\beta = 0.97$, SE = 0.42, $p = 0.021$, OR = 2.64, 95% CI [1.16, 6.01]). In contrast, higher overall fat was associated with reduced odds of being male ($\beta = – 0.55$, SE = 0.19, $p = 0.004$, OR = 0.58, 95% CI [0.40, 0.84]). WHR, WC, and visceral fat were not statistically significant predictors. All data are presented in Table S7.

Table S7. Estimated relationships between sex and anthropometric parameters in healthy adolescents.

Predictor	β Estimate	SE	95% Confidence Interval		Odds ratio	Z	p
			Lower	Upper			
Intercept	-2.805	14.408	-31.045	25.434	0.0605	-0.195	0.846
BMI	0.970	0.420	0.146	1.793	2.6375	2.308	**0.021**
WHR	3.989	32.109	-58.944	66.922	53.9904	0.124	0.901
WC	-0.171	0.260	-0.681	0.340	0.8430	-0.656	0.512
Visceral fat	0.496	0.626	-0.731	1.722	1.6416	0.792	0.428
Overall fat	-0.550	0.190	-0.923	-0.176	0.5772	-2.886	**0.004**

BMI – body mass index, WHR – waist to hip ratio, and WC – waist circumference. A value of $p < 0.05$ (in bold) was considered statistically significant.

Higher BMI is associated with increased odds of being male, while higher overall fat is associated with decreased odds of being male. These results are in line with prior research on sex differences in body composition between sexes. Males typically exhibit greater lean body mass and lower fat mass relative

to females, which contributes to higher BMI values despite lower overall fat percentage [25,26]. The significant inverse relationship between body fat and male sex reflects the influence of sex hormones on fat deposition. Estrogen promotes fat storage, particularly in subcutaneous regions, while testosterone favors lean mass accrual and visceral fat accumulation [27,28]. This helps explain the observed sex-related differences in fat distribution and the predictive power of overall fat in classifying sex. Although WHR, WC, and visceral fat did not reach statistical significance, these variables are nonetheless important in understanding sex-based differences in body composition and disease risk. Males often accumulate more visceral fat, which is associated with metabolic risks, while females store more fat subcutaneously, particularly around the hips and thighs [29]. The wide confidence interval for WHR suggests that further investigation with larger, more balanced samples is needed. In contrast, the model's robust findings for BMI and fat percentage suggest these are reliable, accessible indicators of sex differences in body composition.

References:

1. Hoogeveen, R.C.; Morrison, A.; Boerwinkle, E.; Miles, J.S.; Rhodes, C.E.; Sharrett, A.R.; Ballantyne, C.M. Plasma MCP-1 level and risk for peripheral arterial disease and incident coronary heart disease: Atherosclerosis Risk in Communities study. *Atherosclerosis* **2005**, *183*, 301–307, doi:10.1016/J.ATHEROSCLEROSIS.2005.03.007.
2. Xing, D.; Feng, W.; Miller, A.P.; Weathington, N.M.; Chen, Y.F.; Novak, L.; Blalock, J.E.; Oparil, S. Estrogen modulates TNF-α-induced inflammatory responses in rat aortic smooth muscle cells through estrogen receptor-β activation. *Am. J. Physiol. - Hear. Circ. Physiol.* **2007**, *292*, 2607–2612, doi:10.1152/AJPHEART.01107.2006/ASSET/IMAGES/LARGE/ZH40050774260006.JPEG.
3. Su, C.; Chen, M.; Huang, H.; Lin, J. Testosterone enhances lipopolysaccharide-induced interleukin-6 and macrophage chemotactic protein-1 expression by activating the extracellular signal-regulated kinase 1/2/nuclear factor-κB signalling pathways in 3T3-L1 adipocytes. *Mol. Med. Rep.* **2015**, *12*, 696–704, doi:10.3892/MMR.2015.3401/HTML.
4. Mohammed, A.A.; Abdulla, A.A.; Karam, A.A. Measurement of Inflammation-Related Biomarkers in Different Chronic Kidney Diseases in Humans: Role of Aging and Gender? *IIUM Med. J. Malaysia* **2021**, *20*, 37–43, doi:10.31436/IMJM.V20I4.1827.
5. Jilma-Stohlawetz, P.; Homoncik, M.; Drucker, C.; Marsik, C.; Rot, A.; Mayr, W.R.; Seibold, B.; Jilma, B. Fy phenotype and gender determine plasma levels of monocyte chemotactic protein. *Transfusion* **2001**, *41*, 378–381, doi:10.1046/J.1537-2995.2001.41030378.X.
6. Koenig, J.; Thayer, J.F. Sex differences in healthy human heart rate variability: A meta-analysis. *Neurosci. Biobehav. Rev.* **2016**, *64*, 288–310. doi: 10.1016/j.neubiorev.2016.03.007.
7. Abhishekh, H.A.; Nisarga, P.; Kisan, R.; Meghana, A.; Chandran, S.; Trichur Raju; Sathyaprabha, T.N. Influence of age and gender on autonomic regulation of heart. *J. Clin. Monit. Comput.* **2013**, *27*, 259–264, doi:10.1007/S10877-012-9424-3.
8. Jarczok, M.N.; Kleber, M.E.; Koenig, J.; Loerbroks, A.; Herr, R.M.; Hoffmann, K.; Fischer, J.E.; Benyamini, Y.; Thayer, J.F. Investigating the associations of self-rated health: heart rate variability is more strongly associated than inflammatory and other frequently used biomarkers in a cross sectional occupational sample. *PLoS One* **2015**, *10*, e0117196, doi:10.1371/JOURNAL.PONE.0117196.
9. Bobkowski, W.; Stefaniak, M.E.; Krauze, T.; Gendera, K.; Wykretowicz, A.; Piskorski, J.; Guzik,

P. Measures of Heart Rate Variability in 24-h ECGs Depend on Age but Not Gender of Healthy Children. *Front. Physiol.* **2017**, *8*, 311, doi:10.3389/FPHYS.2017.00311.

10. Faulkner, M.S.; Hathaway, D.; Tolley, B. Cardiovascular autonomic function in healthy adolescents. *Hear. Lung J. Acute Crit. Care* **2003**, *32*, 10–22, doi:10.1067/MHL.2003.6.

11. Cysarz, D.; Linhard, M.; Edelhäuser, F.; Längler, A.; van Leeuwen, P.; Henze, G.; Seifert, G. Unexpected Course of Nonlinear Cardiac Interbeat Interval Dynamics during Childhood and Adolescence. *PLoS One* **2011**, *6*, e19400, doi:10.1371/JOURNAL.PONE.0019400.

12. de Zambotti, M.; Javitz, H.; Franzen, P.L.; Brumback, T.; Clark, D.B.; Colrain, I.M.; Baker, F.C. Sex- and Age-Dependent Differences in Autonomic Nervous System Functioning in Adolescents: Autonomic functioning in adolescence. *J. Adolesc. Health* **2017**, *62*, 184, doi:10.1016/J.JADOHEALTH.2017.09.010.

13. Silvetti, M.S.; Drago, F.; Ragonese, P. Heart rate variability in healthy children and adolescents is partially related to age and gender. *Int. J. Cardiol.* **2001**, *81*, 169–174, doi:10.1016/S0167-5273(01)00537-X.

14. Koenig, J.; Rash, J.A.; Campbell, T.S.; Thayer, J.F.; Kaess, M. A meta-analysis on sex differences in resting-state vagal activity in children and adolescents. *Front. Physiol.* **2017**, *8*, 582, doi: 10.3389/fphys.2017.00582.

15. Loomba-Albrecht, L.A.; Styne, D.M. Effect of puberty on body composition. *Curr. Opin. Endocrinol. Diabetes. Obes.* **2009**, *16*, 10–15, doi:10.1097/MED.0B013E328320D54C.

16. Taylor, R.W.; Grant, A.M.; Williams, S.M.; Goulding, A. Sex differences in regional body fat distribution from pre- to postpuberty. *Obesity (Silver Spring).* **2010**, *18*, 1410–1416, doi:10.1038/OBY.2009.399.

17. Fernández-Vallejo, B.; Monteagudo, F.J.; Romero, L.; Aznárez, M.I.L.; Cobas, M. del C.R.; Pérez-Martínez, L. Cross-Sectional Analysis of IL-6, TNF-α, Adiponectin, Leptin, and Klotho Serum Levels in Relation to BMI Among Overweight and Obese Children Aged 10–14 in La Rioja, Spain. *Children* **2025**, *12*, 89, doi:10.3390/CHILDREN12010089/S1.

18. Klein, S.L.; Flanagan, K.L. Sex differences in immune responses. *Nat. Rev. Immunol.* **2016**, *16*, 626–638, doi:10.1038/NRI.2016.90.

19. Straub, R.H. The complex role of estrogens in inflammation. *Endocr. Rev.* **2007**, *28*, 521–574, doi: 10.1210/er.2007-0001.

20. Losordo, D.W.; Isner, J.M. Estrogen and angiogenesis: A review. *Arterioscler. Thromb. Vasc. Biol.* **2001**, *21*, 6–12, doi:10.1161/01.ATV.21.1.6/ASSET/62669946-8E98-4C0D-9730-FE5D424E0478/ASSETS/GRAPHIC/HQ0111547001.JPEG.

21. Pennell, L.M.; Galligan, C.L.; Fish, E.N. Sex affects immunity. *J. Autoimmun.* **2012**, *38*, J282-291, doi:10.1016/J.JAUT.2011.11.013.

22. Umetani, K.; Singer, D.H.; McCraty, R.; Atkinson, M. Twenty-four hour time domain heart rate variability and heart rate: relations to age and gender over nine decades. *J. Am. Coll. Cardiol.* **1998**, *31*, 593–601, doi:10.1016/S0735-1097(97)00554-8.

23. Sloan, R.P.; Shapiro, P.A.; Bagiella, E.; Boni, S.M.; Paik, M.; Bigger, J.T.; Steinman, R.C.; Gorman, J.M. Effect of mental stress throughout the day on cardiac autonomic control. *Biol. Psychol.* **1994**, *37*, 89–99, doi:10.1016/0301-0511(94)90024-8.

24. Sztajzel, J. Heart rate variability: a noninvasive electrocardiographic method to measure the autonomic nervous system. *Swiss Med. Wkly.* **2004**, *134*, 514–522, doi:10.4414/SMW.2004.10321.

25. Wells, J.C.K. Sexual dimorphism of body composition. *Best Pract. Res. Clin. Endocrinol. Metab.* **2007**, *21*, 415–430, doi:10.1016/J.BEEM.2007.04.007.

26. Kyle, U.G.; Genton, L.; Hans, D.; Karsegard, L.; Slosman, D.O.; Pichard, C. Age-related differences in fat-free mass, skeletal muscle, body cell mass and fat mass between 18 and 94 years. *Eur. J. Clin. Nutr.* **2001**, *55*, 663–672, doi:10.1038/SJ.EJCN.1601198.

27. Toth, M.J.; Tchernof, A.; Sites, C.K.; Poehlman, E.T. Menopause-related changes in body fat distribution. *Ann. N. Y. Acad. Sci.* **2000**, *904*, 502–506, doi:10.1111/J.1749-6632.2000.TB06506.X.

28. Blaak, E. Gender differences in fat metabolism. *Curr. Opin. Clin. Nutr. Metab. Care* **2001**, *4*, 499–502, doi:10.1097/00075197-200111000-00006.

29. Karastergiou, K.; Smith, S.R.; Greenberg, A.S.; Fried, S.K. Sex differences in human adipose tissues - the biology of pear shape. *Biol. Sex Differ.* **2012**, *3*, 13, doi:10.1186/2042-6410-3-13.
